# Progressive plasterer’s pneumoconiosis complicated by fibrotic interstitial pneumonia: a case report

**DOI:** 10.1186/s12890-018-0776-4

**Published:** 2019-01-07

**Authors:** Fumio Kurosaki, Tamiko Takemura, Masashi Bando, Tomonori Kuroki, Toshio Numao, Hiroshi Moriyama, Koichi Hagiwara

**Affiliations:** 1 Division of Pulmonary Medicine, Department of Medicine, Jichi Medical University, 3311-1 Yakushiji, Shimotsuke, Tochigi, 329-0498 Japan; 2 Department of Pulmonary Medicine, National Hospital Organization Utsunomiya National Hospital, 2160 Shimo-Okamoto, Utsunomiya, Tochigi, 329-1193 Japan; 3 Department of Pathology, Japanese Red Cross Medical Center, 4-1-22 Hiroo, Shibuya-ku, Tokyo, 150-8935 Japan; 4 Division of Respiratory Medicine, Graduate School of Medical and Dental Sciences, Niigata University, 1-757 Asahimachi-dori, Niigata, 951-8510 Japan

**Keywords:** Plasterer, Pneumoconiosis, Usual interstitial pneumonia, Elemental analysis

## Abstract

**Background:**

Although the prevalence of pneumoconiosis has been decreasing due to improvements in working conditions and regular health examinations, occupational hygiene measures are still being established. Plasterers encounter a number of hazardous materials that may be inhaled in the absence of sufficient protection.

**Case presentation:**

A 64-year-old man who plastered without any dust protection for more than 40 years was referred to our hospital with suspected interstitial pneumonia. Mixed dust pneumoconiosis and an unusual interstitial pneumonia (UIP) pattern with fibroblastic foci were diagnosed by video-assisted thoracoscopic surgery, and an elemental analysis detected elements included in plaster work materials. Despite the cessation of plaster work and administration of nintedanib, the patient developed advanced respiratory failure.

**Conclusion:**

Plasterers are at an increased risk of pneumoconiosis and may have a poor prognosis when complicated by the UIP pattern. Thorough dust protection and careful monitoring are needed.

**Electronic supplementary material:**

The online version of this article (10.1186/s12890-018-0776-4) contains supplementary material, which is available to authorized users.

## Background

With energy transition from coal to oil and nuclear power, coal mines completely disappeared by the early first decade of the 2000s in Japan. Furthermore, improvements in industrial hygiene and vocational education have protected workers against mine dust exposure; therefore, pneumoconiosis is becoming an uncommon disease [1]. Although the prevalence of pneumoconiosis was originally high among plasterers [2], there have been few case reports of pneumoconiosis since the use of asbestos was prohibited. We herein present a case of pneumoconiosis in a plasterer diagnosed by video-assisted thoracoscopic surgery (VATS) who developed progressive respiratory failure without effective treatment. VATS specimens showed mixed dust pneumoconiosis (MDP) and an unusual interstitial pneumonia (UIP) pattern, the cause of which was identified as plaster work by an elemental analysis. Therefore, plasterers need to take proper countermeasures for dust prevention and undergo regular examinations.

## Case presentation

**Fig. 1:**
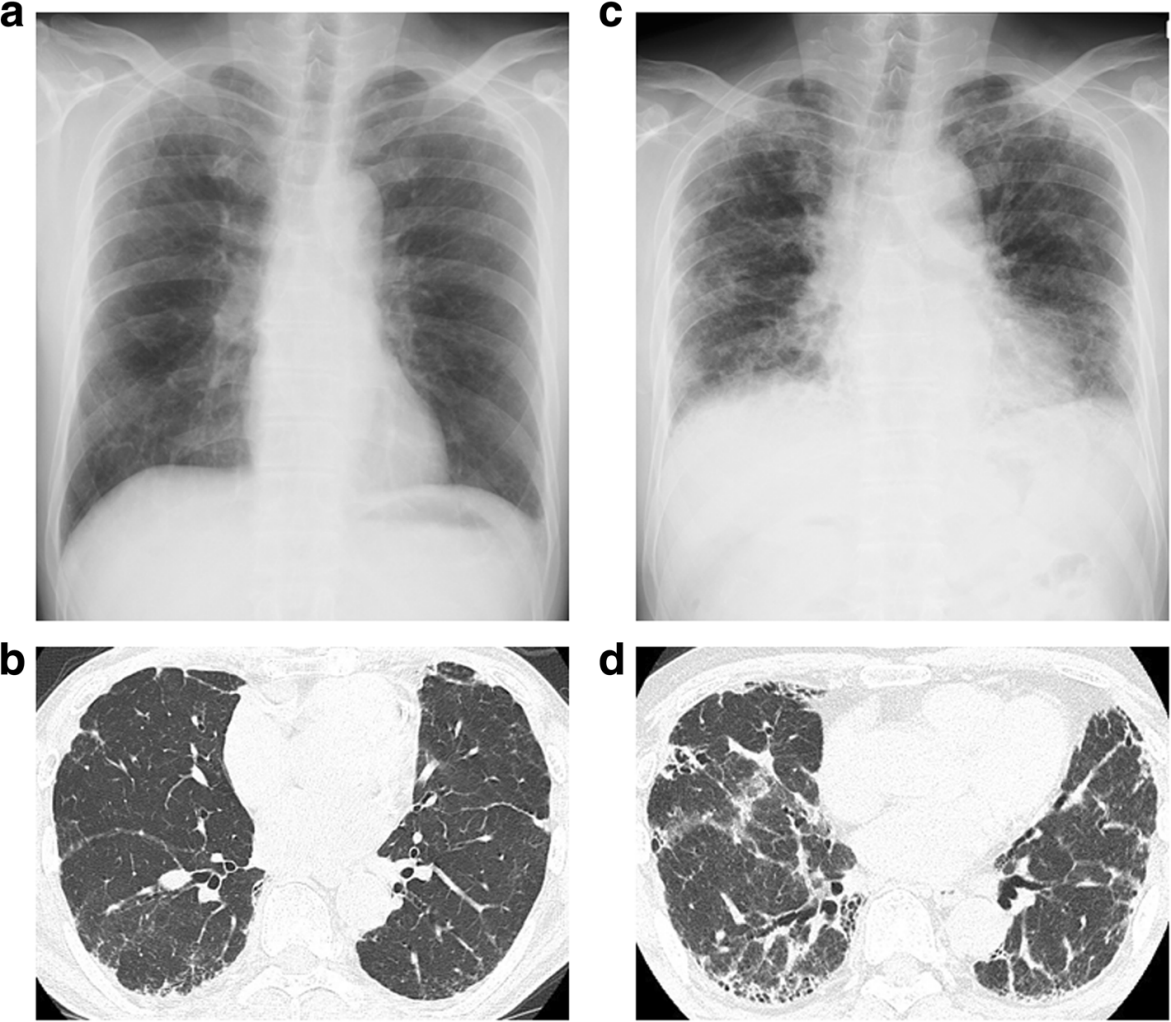
Chest X-ray and HRCT findings in 2013 (panels **a** and **b**) and 2018 (panels **c** and **d**). Both examinations revealed reticular opacities in the subpleural regions that progressed over the course of 5 years and were associated with a markedly reduced lung volume

**Fig. 2:**
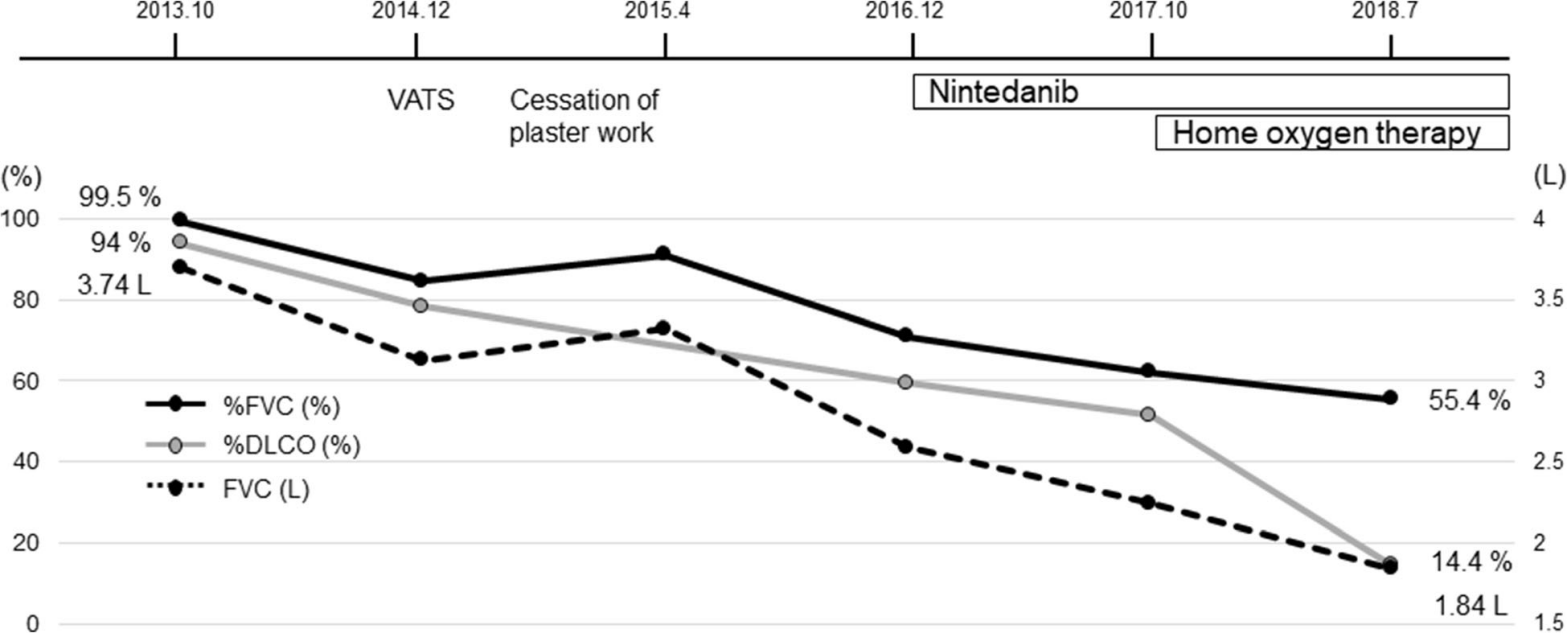
Clinical course and changes in pulmonary function test results. Although the cessation of plaster work was effective temporarily, the patient developed progressive respiratory failure without effective treatment

A 64-year-old non-smoking Japanese man was referred to our hospital with suspected interstitial pneumonia in a health examination in 2013. He had a slightly dry cough with no desaturation. He had been a plasterer for more than 40 years without appropriate protective equipment. Chest auscultation revealed slight bilateral inspiratory fine crackles in the bilateral lower lung zones. A chest X-ray film showed enlarged hilar lymph nodes and mild reticular opacities, mainly in the upper to middle lung fields of both lungs (Fig. 1a). The results of a chest high-resolution computed tomography (HRCT) scan suggested a predominantly subpleural distribution of irregular linear opacities and reticulonodular shadows with interlobular septal thickening in both lung fields (Fig. 1b). Pulmonary function tests were close to normal (Fig. 2) and a six-minute walking test performed on admission was also normal.

**Fig. 3:**
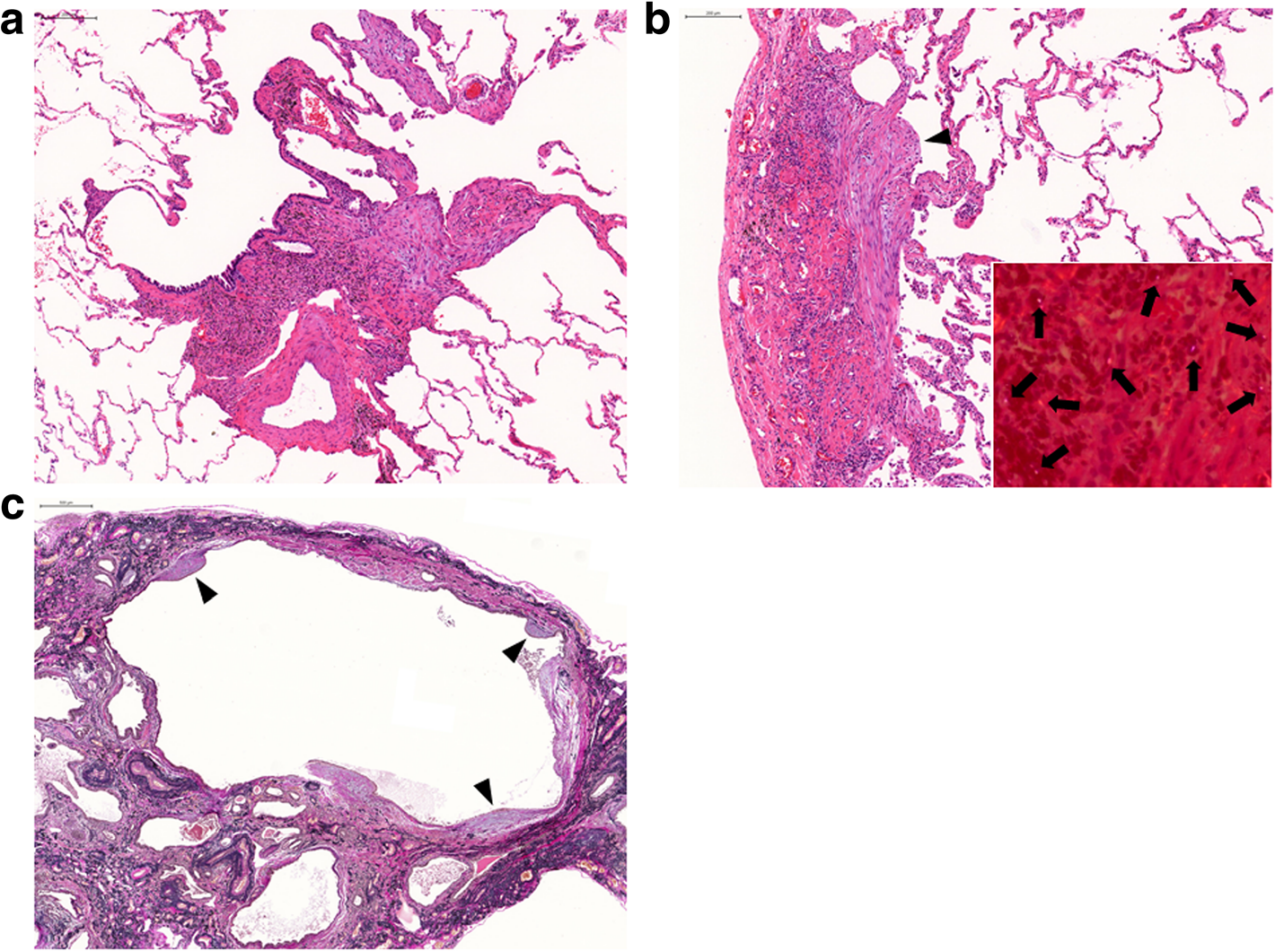
Histopathological findings from surgical lung biopsy specimens. **a** Hematoxylin and eosin (HE) staining of the right upper lung lobe S2 specimen revealed dense fibrosis with mononuclear cell infiltration and inorganic dust particles around the respiratory bronchioles, consistent with mixed dust pneumoconiosis. **b** Another field from the right upper lung lobe S2 specimen showed fibrously thickened interlobular septa and visceral pleura accompanied by fibroblastic foci (black arrowhead). It also identified some birefringent particles (black arrows) by polarized light microscopy (inset). **c** Elastica van Gieson staining of the right lower lung lobe S9 specimen showed extensive honeycomb changes and fibroblastic foci (black arrowheads) within the cystic wall. Scale bars indicate 200 μm

One year later (in 2014), forced vital capacity (FVC) and diffusing capacity of the lungs for carbon monoxide (DLCO) had decreased (Fig. 2), while reticulonodular shadows on HRCT worsened. However, his thoracic symptoms had not deteriorated and his vital signs were stable. In order to establish a diagnosis, VATS was performed from the right S2 segment of the upper lung lobe and the right S9 segment of the lower lung lobe (Fig. 3). Dense fibrosis with mononuclear cell infiltration and inorganic dust particles around the respiratory bronchioles was observed in the upper lung lobe S2 segment, which was consistent with MDP (Fig. 3a). Furthermore, fibrously thickened interlobular septa and visceral pleura accompanied by dust, including some birefringent particles suggestive of silicates, and fibroblastic foci were detected within these lesions (Fig. 3b). Extensive honeycomb changes with dilated bronchioles and parenchymal collapse as well as fibroblastic foci within the cystic wall were observed in the lower lung lobe S9 segment (Fig. 3c). Asbestos fibers and asbestos bodies were not found in either upper lung lobe S2 or lower lung lobe S9 specimens. An electron probe microanalysis (EPMA) primarily detected silicon (Si), aluminum (Al), and iron (Fe) in the S2 and S9 areas (Additional file 1: Figure S1).

Based on these results, we diagnosed the patient with pneumoconiosis with the UIP pattern and monitored him without any medication because he quit his job. Although his symptoms and pulmonary function tests transiently improved, he had a dry cough with exertional dyspnea, and his pulmonary function test results markedly deteriorated (in 2016, Fig. 2). We administered nintedanib with the expectation of an anti-fibrotic effect; however, the treatment was not effective, and he currently requires home oxygen therapy (Fig. 2). A recent chest X-ray image (Fig. 1c) showed a markedly smaller lung volume than that in the initial image as well as the progression of lung fibrosis. A chest CT scan also showed the progression of fibrotic changes in subpleural regions (Fig. 1d).

## Discussion and conclusions

This case suggests that plasterers are at an increased risk of MDP due to occupational exposure and that this condition may be progressive when complicated by the UIP pattern.

Plasterers perform a number of tasks including the interior and exterior plastering of drywall, cement, stucco, and stone imitation [3]. They are exposed to many potentially toxic substances, such as cement dust, silicate, fiberglass, talc, and asbestos [3]. Nevertheless, some plasterers continue to work without sufficient protection against dust exposure. Our patient was a plasterer for more than 40 years and was continually exposed to dust during that time. His VATS specimens showed mixed dust fibrosis, fibrously thickened interlobular septa, and visceral pleura, consistent with pneumoconiosis. These lesions were located along the intrapulmonary lymph nodes [4, 5], which indicated long-term mine dust exposure. Furthermore, a large amount of dust was deposited within the fibrotic lesions and an elemental analysis mainly detected Si, Al, and Fe. These elements are included in cement and vermiculite, which is used for interior decoration, and long-term dust inhalation may damage the lung architecture; therefore, plasterers are at risk of pneumoconiosis.

Occupational exposure may contribute to the development of pulmonary fibrosis [6]. Asbestosis is complicated by interstitial pneumonia [6]. Previous studies suggested that non-asbestos pneumoconiosis is also a complication in up to 30% of cases, with most having the UIP pattern [7, 8]. These cases may be more closely related to MDP than typical silicosis [9] because MDP is caused by exposure to fibrogenic mine dust as well as silica [10]. In our case, with the deposition of a large amount of dust, honeycomb changes and fibroblastic foci were observed in a lower lobe, which indicated fibrogenesis and are characteristic features of UIP [11]. In contrast to ordinary UIP, fibroblastic foci are uncommon and there are a limited number of reports of these foci among cases of interstitial pneumonia complicated by pneumoconiosis [12–17]; these patients were exposed to silicates [12, 13, 16, 17], asbestos [12, 15, 16], and hard metals, such as tungsten [14, 16]. Fibroblastic foci are composed of the accumulation of fibroblasts or myofibroblasts, which play a critical role in the progression of pulmonary fibrosis, and correlate with disease progression and a poor prognosis [18].

There are no curative therapies for pneumoconiosis and supportive therapy is the best therapeutic option; management is limited to the avoidance of further dust exposure, symptomatic therapy, such as bronchodilators for the treatment of airflow obstruction, and the prevention of infection [19, 20]. In our case, the cessation of plastering provided only a temporary effect, the administration of nintedanib was ineffective, and he currently uses supplemental oxygen. Due to the lack of pharmacotherapeutic evidence for pneumoconiosis-related interstitial pneumonia, the administration of corticosteroids is sometimes attempted [20]. However, large, randomized clinical trials have not yet been performed, and, thus, the administration of corticosteroids is not recommended, except for some granulomatous cases such as berylliosis [21] and hypersensitive pneumonitis caused by dust [22]. On the other hand, the efficacy of anti-fibrotic drugs, including pirfenidone and nintedanib used in the present case, is unknown and, thus, further detailed studies are warranted.

In summary, we herein present an advanced case of pneumoconiosis in a plasterer. Construction workers, including plasterers, inhale a number of toxic substances and are at an increased risk of MDP. They may develop progressive respiratory failure without effective treatment when complicated by interstitial pneumonia. Appropriate countermeasures for dust prevention and careful monitoring are needed to prevent the development of these lung diseases.

## Additional file

Additional file 1: **Figure S1**. Images of light micrographs and electron probe microanalysis (EPMA). (a-e) A right upper lung lobe S2 lesion shows mixed dust pneumoconiosis (a: HE stain; b: silicon [Si]; c: aluminum [Al]; d: iron [Fe]; e: quantitative analysis). (f-j) Another right upper lung lobe S2 lesion shows fibrously thickened interlobular septa and visceral pleura (f: HE stain; g: Si; h: Al; i: Fe; j: quantitative analysis). (k-o) A right lower lung lobe S9 lesion shows honeycomb changes (k: HE stain; l: Si; m: Al; n: Fe; o: quantitative analysis). (PPTX 2144 kb)

## Abbreviations

Al: Aluminum
DLCO: Diffusing capacity of the lungs for carbon monoxide
EPMA: Electron probe microanalysis
Fe: Iron
FVC: Forced vital capacity
HRCT: High-resolution computed tomography
MDP: Mixed dust pneumoconiosis
Si: Silicon
UIP: Unusual interstitial pneumonia
VATS: Video-assisted thoracoscopic surgery
