# Supplementary figures and images for: Progressive plasterer’s pneumoconiosis complicated by fibrotic interstitial pneumonia: a case report
Source: BMC Pulm Med. 2019 Jan 7;19:6. doi: 10.1186/s12890-018-0776-4 (PMC6323823; doi:10.1186/s12890-018-0776-4)

## Slide 1
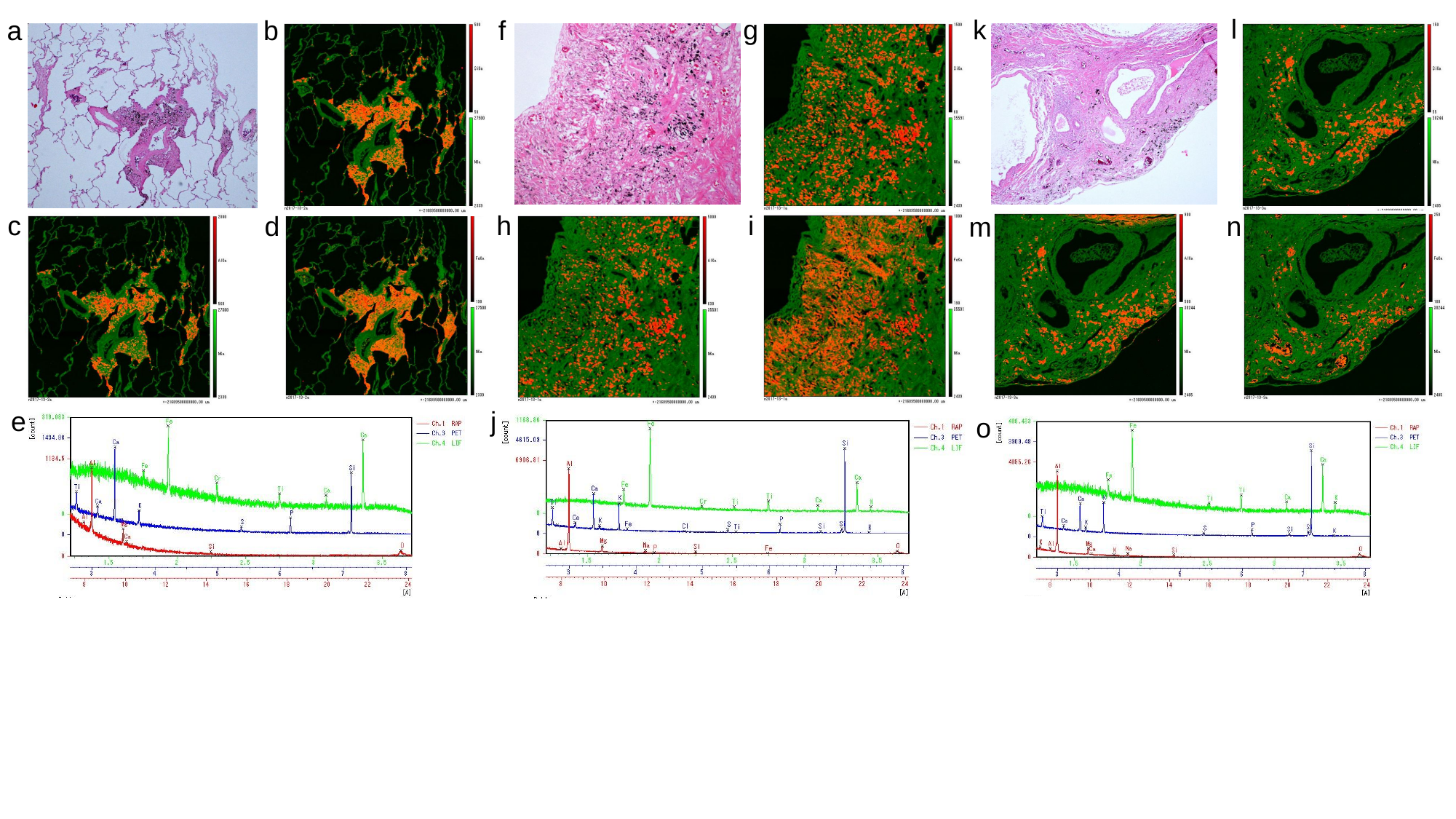

l
g
k
a
b
f
i
c
h
d
n
m
e
j
o

Supplement: Supplementary file 1 — Figure S1. Images of light micrographs and electron probe microanalysis (EPMA). (a-e) A right upper lung lobe S2 lesion shows mixed dust pneumoconiosis (a: HE stain; b: silicon [Si]; c: aluminum [Al]; d: iron [Fe]; e: quantitative analysis). (f-j) Another right upper lung lobe S2 lesion shows fibrously thickened interlobular septa and visceral pleura (f: HE stain; g: Si; h: Al; i: Fe; j: quantitative analysis). (k-o) A right lower lung lobe S9 lesion shows honeycomb changes (k: HE stain; l: Si; m: Al; n: Fe; o: quantitative analysis). (PPTX 2144 kb) [file 12890_2018_776_MOESM1_ESM.pptx]
